# Supplementary material for: Recognition & management of varicella infections and accuracy of antimicrobial recommendations: Case vignettes study in the US
Source: PLoS One. 2022 Jun 24;17(6):e0269596. doi: 10.1371/journal.pone.0269596 (PMC9231738; doi:10.1371/journal.pone.0269596)
Supplement: S1 File — Vignette information and survey file. (DOCX) [file pone.0269596.s001.docx]

**Supplementary Information about the Vignettes**

The first was a non-varicella case/vignette was included to gauge providers ability to distinguish between conditions with symptoms/presentations similar to varicella that need antibiotics. It had been deliberately added to avoid having all varicella cases. The other was a varicella case but removed after expert consultation since the choice of correct treatment was unclear due to the complexity of the case. Further clinical information, not provided in the vignettes, would have been necessary to make an appropriate treatment recommendation.

For vignette#8, after expert consultation, the use of antivirals was deemed unnecessary based on the duration of time (5 days rash) & description of an immunocompetent individual. The 5 days vs 3 days was the critical difference since the AAP Red Book indicates that “Antiviral drugs have a limited window of opportunity to affect the outcome of VZV infection. In immunocompetent hosts, most virus replication has stopped by 72 hours after onset of rash; the duration of replication may be extended in immunocompromised hosts” .[1]

Similarly, for Vignettes#7 again, the 5-day history of rash in a previously healthy 6 year old child meant that the pulmonary complications were more likely secondary bacterial pneumonia rather than primary viral process.[1] It would be most appropriate to hospitalize and start antibiotics. While some physicians may prefer to start antivirals just in case while working the child up for immunodeficiency, so we feel that the appropriate treatment would be hospitalization + antibiotics.

Vignettes Informed Consent and Survey

**Module 1: Eligibility Screener**

*To be presented only if and after respondent agrees to participate in the study.*

1. **What is your degree?**

| 1 | Medical doctor (MD) |
| --- | --- |
| 2 | Doctor of Osteopathic Medicine (DO) |
| 3 | Master’s degree (e.g. nursing) |
| 4 | Doctor of Nursing Practice |
| 5 | PhD in Nursing |
| 6 | None of the above |
| 7 | Don’t know/Not sure |
| 8 | Prefer not to answer |

*If participant states “None of the above”, “Don’t know/Not sure”, or “I prefers not to answer” then they are ineligible for the survey. If provider selects “Medical doctor (MD)” or “Doctor of Osteopathic Medicine (DO)” continue to question 2. If provider selects “Master’s degree (e.g. nursing)” or “Doctor of Nursing Practice” or “PhD in Nursing” skip to Question 7.*

***Doctor specific screener (based on response to Q1)***

1. **Are you currently licensed in the state you practice in to prescribe medicine?**

| 1 | Yes |
| --- | --- |
| 2 | No |
| 3 | Don’t know/Not sure |
| 4 | I prefer not to answer |

*If participant states “No”, “Don’t know/Not sure”, or “I prefer not to answer” then they are ineligible for the survey.*

1. **Which of the following areas do you currently hold a board-certified specialty in? *Choose all that apply.***

| 1 | Anesthesiology |
| --- | --- |
| 2 | Dermatology |
| 3 | Emergency Medicine |
| 4 | Family Medicine |
| 5 | Internal Medicine |
| 6 | Neurology |
| 7 | Obstetrics & Gynecology |
| 8 | Oncology |
| 9 | Ophthalmology |
| 10 | Orthopedic Surgery |
| 11 | Otolaryngology |
| 12 | Pediatrics |
| 13 | Physical Medicine & Rehabilitation |
| 14 | Preventive Medicine |
| 15 | Psychiatry |
| 16 | Radiology |
| 17 | Surgery |
| 18 | I prefer not to answer |
| 19 | Other, please specify |

*If participant does not state “General Practice/Internal Medicine”, “Family Medicine” or “Pediatrics” or refuses to answer then they are ineligible for the survey. If provider selects ‘12’ “Pediatrics” proceed to question 4. If provider selects “General Practice/Internal Medicine” skip to question 5.*

1. **What is your pediatric sub-specialty?**

| 1 | Adolescent Medicine |
| --- | --- |
| 2 | Pediatric Cardiology |
| 3 | Child Abuse Pediatrics |
| 4 | Pediatric Critical Care Medicine |
| 5 | Developmental-Behavioral Pediatrics |
| 6 | Pediatric Emergency Medicine |
| 7 | Pediatric Endocrinology |
| 8 | Pediatric Gastroenterology |
| 9 | Pediatric Hematology-Oncology |
| 10 | Pediatric Hospital Medicine |
| 11 | Pediatric Infectious Diseases |
| 12 | Neonatal-Perinatal Medicine |
| 13 | Pediatric Nephrology |
| 14 | Pediatric Rheumatology |
| 15 | I do not have a sub-specialty |
| 16 | I prefer not to answer |
| 17 | Other, please specify |

1. **Are you currently licensed to practice medicine in the United States?**

| 1 | Yes |
| --- | --- |
| 2 | No |
| 3 | Don’t know/Not sure |
| 4 | I prefer not to answer |

*If participant states “No”, “Don’t know/Not sure”, or “I prefer not to answer” then they are ineligible for the survey.*

1. **In what year did you first obtain your medical license?**

| 1 | Drop down menu ‘1950-2019’ |
| --- | --- |
| 2 | I am not a licensed Provider |
| 3 | I prefer not to answer |

*If the provider answers “I am not a licensed Provider or nurse practitioner” or refuses to answer, the provider is ineligible. If provider selects a valid option from the drop down menu, skip to Question 13.*

***Nursing specific screener (based on response to Q1)***

1. **Please select your nursing license type**

| 1 | Nurse practitioner (NP) |
| --- | --- |
| 2 | Registered nurse (RN) |
| 3 | Licensed practical nurse (LPN) |
| 4 | Certified pediatric nurse (CPN) |
| 6 | None of the above |
| 7 | Don’t know/Not sure |
| 8 | Prefer not to answer |

*If provider selects “Nurse practitioner (NP)” continue to question 8. If provider selects “Registered nurse (RN)” or “Licensed practical nurse (LPN)” or “Certified pediatric nurse (CPN)” or “None of the above” or “Don’t know/Not sure” or “Prefer not to answer” respondent is not eligible for the survey.*

1. **Are you currently licensed in the state you practice in to prescribe medicine?**

| 1 | Yes |
| --- | --- |
| 2 | No |
| 3 | Don’t know/Not sure |
| 4 | I prefer not to answer |

*If participant states “No”, “Don’t know/Not sure”, or “I prefer not to answer” then they are ineligible for the survey.*

1. **Which of the following areas do you currently hold a board-certified specialty in? *Choose all that apply.***

| 1 | Anesthesiology |
| --- | --- |
| 2 | Dermatology |
| 3 | Emergency Medicine |
| 4 | Family Medicine |
| 5 | Internal Medicine |
| 6 | Neurology |
| 7 | Obstetrics & Gynecology |
| 8 | Oncology |
| 9 | Ophthalmology |
| 10 | Orthopedic Surgery |
| 11 | Otolaryngology |
| 12 | Pediatrics |
| 13 | Physical Medicine & Rehabilitation |
| 14 | Preventive Medicine |
| 15 | Psychiatry |
| 16 | Radiology |
| 17 | Surgery |
| 18 | I prefer not to answer |
| 19 | Other, please specify |

*If participant does not state “General Practice/Internal Medicine”, “Family Medicine”, or “Pediatrics” or refuses to answer then they are ineligible for the survey. If provider selects ‘12’ “Pediatrics” proceed to question 10. If provider selects “General Practice/Internal Medicine” skip to question 11.*

1. **What is your pediatric sub-specialty? *Choose all that apply*.**

| 1 | Adolescent Medicine |
| --- | --- |
| 2 | Pediatric Cardiology |
| 3 | Child Abuse Pediatrics |
| 4 | Pediatric Critical Care Medicine |
| 5 | Developmental-Behavioral Pediatrics |
| 6 | Pediatric Emergency Medicine |
| 7 | Pediatric Endocrinology |
| 8 | Pediatric Gastroenterology |
| 9 | Pediatric Hematology-Oncology |
| 10 | Pediatric Hospital Medicine |
| 11 | Pediatric Infectious Diseases |
| 12 | Neonatal-Perinatal Medicine |
| 13 | Pediatric Nephrology |
| 14 | Pediatric Rheumatology |
| 15 | I do not have a sub-specialty |
| 16 | I prefer not to answer |
| 17 | Other, please specify |

1. **Is your nursing or other advanced degree from an accredited school in the United States?**

| 1 | Yes |
| --- | --- |
| 2 | No |
| 3 | Don’t know/Not sure |
| 4 | I prefer not to answer |

*If participant states “No”, “Don’t know/Not sure”, or “I prefer not to answer” then they are ineligible for the survey.*

1. **In what year did you first obtain your nurse practitioner’s license in the state you currently practice in?**

| 1 | Drop down menu ‘1950-2019’ |
| --- | --- |
| 2 | I am not a licensed Provider or nurse practitioner |
| 3 | I prefer not to answer |

*If the provider answers “I am not a licensed Provider or nurse practitioner ” or refuses to answer, the provider is ineligible.*

***All provider screener continues***

**Approximately what proportion of your time is spent in a clinical setting (e.g. treating patients, writing notes, etc. as opposed to conducting research, teaching in a classroom setting, managing staff, etc.), on average in the last year?**

| 1 | 0-24% |
| --- | --- |
| 2 | 25-49% |
| 3 | 50-74% |
| 4 | 75-100% |
| 5 | Don’t know/Not sure |
| 6 | I prefer not to answer |

If the provider answers “0-24%”, “24-49%”, “I currently do not practice”, “Don’t know/Not Sure”, or “I prefer not to answer” the provider is ineligible.

**14. During a typical month, on average how many pediatric patients do you prescribe or administer vaccines to?**

| 1 | None |
| --- | --- |
| 2 | 1-4 patients |
| 3 | 5-10 patients |
| 4 | 11-20 patients |
| 5 | 21-50 patients |
| 6 | More than 50 patients |
| 7 | Don’t know/Not sure |
| 8 | I prefer not to answer |

If the provider answers “None”, “Don’t know/Not Sure”, or “I prefer not to answer” the provider is ineligible.

**Module 2: Case Vignettes**

*To be presented only if and after respondent completes Module 1.*

You will be presented with a series of hypothetical patient cases/vignettes. Using your expertise, please evaluate the vignettes and identify the diagnosis and treatment plan you would recommend/prescribe, including medication utilization, to the best of your ability. While in real world practice you may consider other contextual factors such as perceived ability of patient/family to be adherent to treatment, the impact of treatment on family/child, and so on, please consider the material contained in the case description only in selecting your response.

**1. Case Vignette 1**

A 3.5-year-old child presented with a 3-day history of pruritic rash which consisted of ~300 vesicular lesions (see photo below), and a 4-day history of fatigue, loss of appetite, and headache. The lesions originally appeared on the child’s trunk/torso and spread to the face and limbs. There was no history of fever. On examination, vital signs were normal. Examination of the eyes, heart, lungs, abdomen, skin, and nervous system yielded no pathologic findings. Growth and development were otherwise normal. All of the child’s immunizations were not up to date according to the recommended US child vaccine schedule. The child attends daycare, but no known exposure to common pathogens was reported.


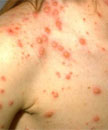


Source: [http://www.vaccineinformation.org/photos/variaap001.jpgCdc-imageExternal](http://www.vaccineinformation.org/photos/variaap001.jpg)
Copyright: American Academy of Pediatrics

**Based on the case description above, what do you feel is the most likely diagnosis?**

**What treatment strategy would you recommend and/or prescribe?**

***14a. Based on the case description above, what do you feel is the most likely primary diagnosis? [PN:Select one]***

- Varicella [SHOW sub-options if Varciella is selected]
  - Varicella without complication
  - Varicella with complications
  - Varicella meningitis
  - Varicella encephalitis and encephalomyelitis
  - Varicella myelitis
  - Varicella pneumonia
  - Varicella keratitis
  - Other varicella complications
- Hand foot and mouth disease
- Bacterial infection NOS
- Pruritus, unspecified
- Impetigo
- Scabies
- Poison oak/ivy
- Molluscum contagiosum
- Folliculitis
- Conjunctivitis
- Cellulitis
- Other

***14b. Based on the case description above, what treatment strategy would you recommend and/or prescribe? [PN:Select all that apply]***

- Supportive care [SHOW sub-options below if Supportive care is selected] [PN:Select all that apply]
  - Acetaminophen
  - Ibuprofen
  - Calamine lotion
  - Colloidal oatmeal bath
  - Zinc oxide
  - Topical antihistamines
  - Topical corticosteroids
  - Topical antifungal
  - Antiseptic cleansers (hydrogen peroxide, chlorhexidine etc.)
  - Topical antibiotics
  - Warm compresses to relieve itching and pain
  - Other supportive care
- Administer varicella vaccine
- Administer pneumococcal vaccine
- Administer varicella immunoglobulin
- If contracted with chicken pox and at risk of complications due to virus reactivation
- Other catch-up vaccines
- Treatment with antivirals [SHOW sub-options below if Treatment with antivirals is selected] *[PN:Select all that apply]*
  - Acyclovir P.O.
  - Acyclovir I.V.
  - Valacyclovir P.O.
  - Other antiviral
- Treatment with antibiotics [SHOW sub-options below if Treatment with antibiotics is selected] [PN:Select all that apply]
  - Aminoglycosides
  - Carbapenems
  - Cephalosporins
  - Fluoroquinolones
  - Macrolides
  - Monobactams
  - Penicillin
  - Sulfonamides/trimethoprim
  - Tetracyclines
  - Other antibiotic(s)
- Hospitalization: [SHOW sub-options below Hospitalization is selected] [PN:Select all that apply]
  - Manage as outpatient
  - Admit for inpatient treatment
- Laboratory tests [SHOW sub-options below Laboratory tests is selected] [PN:Select all that apply]
  - CBC
  - Polymerase chain reaction (PCR) assay for varicella
  - Direct fluorescent antibody (DFA) assay for varicella
  - Viral culture
  - Serology (IgG) for varicella
  - Capture IgM
  - Tzanck smear
  - Blood culture
  - Wound culture
  - Sputum culture
  - Complete metabolic panel
- Imaging [SHOW sub-options below imaging is selected]
  - Chest X-ray
  - MRI of an affected extremity/abdomen/chest
  - CT scan of the affected area
- Other

**2. Case Vignette 2**

A 5-year-old child presented with a 1-day history of pruritic rash, which consisted of approximately 35 papular lesions (see photo below), malaise, and high-grade fever that began at the same time as the rash. The lesions originally appeared on the child’s abdomen and spread to the arms and face. There was no history of fever, headache, or vomiting before the onset of symptoms 1-day prior. On examination, vital signs were normal. Examination of the eyes, heart, lungs, abdomen, skin, and nervous system revealed no abnormal findings except for high fever (101° F). Growth and development of the child were otherwise normal. The child has not yet received the vaccines as recommended by the ACIP US vaccination schedule as recommended for children between the ages of 4 and 6. The child attends school and was exposed to another child with varicella-like symptoms approximately 14 days earlier. No other known exposures to other pathogens was reported.


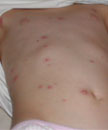


Sources: CDC and Varicella Active Surveillance Project

**Based on the case description above, what do you feel is the most likely diagnosis?**

**What treatment strategy would you recommend and/or prescribe?**

**3. Case Vignette 3**

A 7-year-old child presented with a 3-day history of pruritic rash, which consisted of approximately 250 papular lesions (see photo below), abdominal pain, loss of appetite, and high-grade fever that began 2 days after the rash erupted. The rash originally appeared on the child’s chest and spread to limbs and face. Several lesions on the child’s face were enlarged, purulent, and erythematous. On examination, aside from a continued fever of 101.5° F, vital signs were normal. Examination of the eyes, heart, lungs, abdomen, skin, and nervous system yielded no pathologic findings. Growth and development were otherwise normal. The family has recently moved to this country and the status of the childhood vaccinations was unknown. The child had no known exposure to measles, shingles, varicella, hand foot and mouth disease, scabies, or poison ivy/oak.


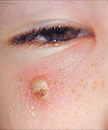


Source: CDC.

**Based on the case description above, what do you feel is the most likely diagnosis?**

**What treatment strategy would you recommend and/or prescribe?**

**4. Case Vignette 4**

A 3-year-old child presented with a 1-day history of pruritic rash, which consisted of approximately 150 papular lesions (see photo below), vomiting, loss of appetite, and high-grade fever that began at approximately the same time as the rash. The rash originally appeared on the child’s chest and back. The child has a history of asthma and is currently on 28mg/day oral prednisone. The last asthma exacerbation was 3 days prior to the onset of rash and other symptoms. On examination, the child’s breathing rate and temperature (101° F) were found to be elevated. Growth and development were otherwise normal. All of the child’s immunizations were up to date according to the recommended US child vaccine schedule except varicella and MMR, given the history of steroid use. The child had no known exposures to infectious disease agents, except for exposure to shingles by their 67-year-old grandfather 7 days prior to the onset of the child’s symptoms.


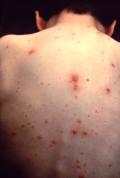


Source: Public Health Image Library

**Based on the case description above, what do you feel is the most likely diagnosis?**

**What treatment strategy would you recommend and/or prescribe?**

**5. Case Vignette 5**

A 10-month-old child presented with a 2-day history of pruritic rash, which consisted of approximately 250 vesicular lesions, fatigue, and a high-grade fever. The fever and fatigue began along with rash. The lesions originally appeared on the child’s back and have begun spreading to their limbs. On examination, the child’s temperature was 99.5° F and other vital signs were normal. Examination of the eyes, heart, lungs, abdomen, skin, and nervous system yielded no pathologic findings. Growth and development were otherwise normal. All of the child’s immunizations were up to date according to the recommended US child vaccine schedule. The child attends day care and had likely been exposed to varicella from a playmate 12 days prior to the onset of their symptoms. No known exposures to other pathogens were reported.


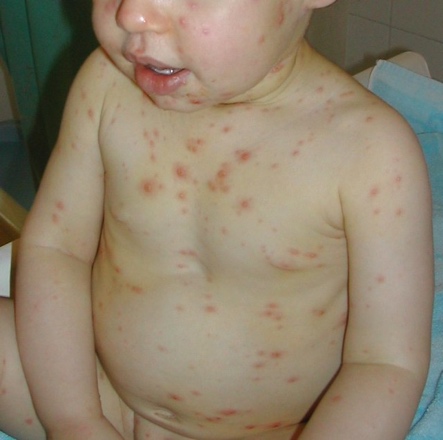


Source: Noj Han, [Flickr](https://www.flickr.com/photos/nojhan/472561609)

**Based on the case description above, what do you feel is the most likely diagnosis?**

**What treatment strategy would you recommend and/or prescribe?**

**6. Case Vignette 6**

A 15-year-old previously healthy adolescent presented with a 3-day history of pruritic rash, which consisted of approximately 300 vesicular lesions, loss of appetite, fatigue, and headache. The lesions originally appeared on the child’s back and then spread to their abdomen, face, and limbs. On examination, vital signs were normal. Examination of the eyes, heart, lungs, abdomen, skin, and nervous system yielded no pathologic findings. Growth and development were normal. The patient and their siblings received a personal belief vaccination exemption. The adolescent’s 7-year-old sibling had varicella infection, which presented 10 days before the onset of the 15-year-old’s symptoms. The child had no known exposure to measles, shingles, hand foot and mouth disease, scabies, or poison ivy/oak.


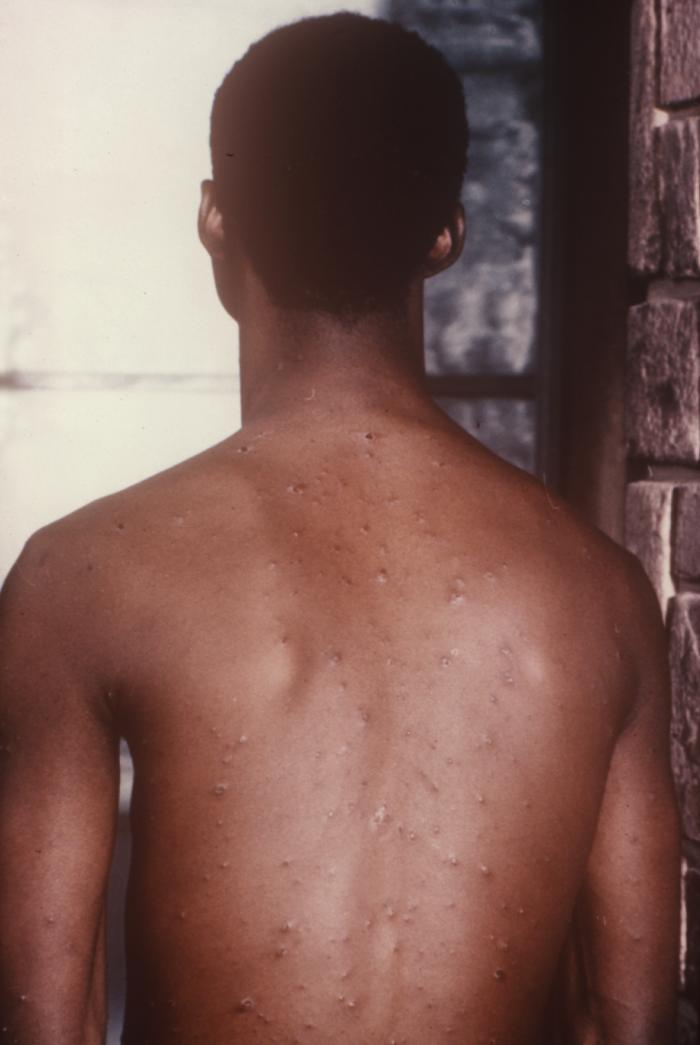


Source: Public Health Image Library

**Based on the case description above, what do you feel is the most likely diagnosis?**

**What treatment strategy would you recommend and/or prescribe?**

**7. Case Vignette 7**

A 6-year-old previously healthy child presented with a 5-day history of pruritic rash, which consisted of approximately 350 vesicular lesions, loss of appetite, fatigue, and chest pain. The patient developed a high-grade fever and cough 4 days after the onset of rash symptoms. On examination, the child’s temperature was 102° F. The child was unable to tolerate oral intake, had a respiratory rate < 50/minute, and decreased oxygen saturation (65%). Auscultation of lungs revealed crackles in lower lobes of lungs. Growth and development were otherwise normal. All of the child’s immunizations were not up to date according to the recommended US child vaccination schedule: the child missed the second doses of the MMR and varicella vaccines. The child had no known exposure to hand foot and mouth disease, scabies, or poison ivy/oak.


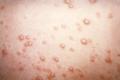


Source: Public Health Image Library

**Based on the case description above, what do you feel is the most likely diagnosis?**

**What treatment strategy would you recommend and/or prescribe?**

**8. Case Vignette 8**

A 14-year-old previously healthy adolescent presented with a 5-day history of pruritic rash, which consisted of approximately 400 vesicular lesions, loss of appetite, fatigue, headache, and a low-grade fever. On examination, vital signs were normal. Examination of the eyes, heart, lungs, abdomen, skin, and nervous system yielded no pathologic findings. Growth and development were otherwise normal. The child’s immunizations were not up to date according to the recommended US child vaccine schedule (they had a religious exemption and declined all vaccinations after 12 months of age). The child attends school, but no known exposure to bacterial or viral infections was reported.


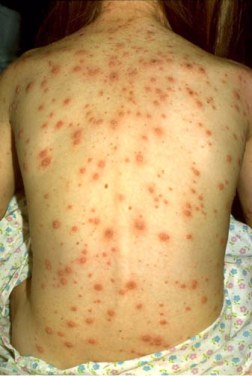


Source: CDC

**Based on the case description above, what do you feel is the most likely diagnosis?**

**What treatment strategy would you recommend and/or prescribe?**

**9. Case Vignette 9 (NOT INCLUDED IN ANALYSIS SINCE IT IS NOT A VARICELLA VIGNETTE)**

A 6-year-old child presented with a 4-day history of pruritic rash, which consisted of approximately 150 vesicular lesions (see photo below). The rash originally appeared on the child’s back and has remained concentrated there. On examination, the child’s vital signs were normal. Growth and development were otherwise normal. The child doesn’t attend school and vaccination status is unknown. Additionally, the child had no known exposure to bacterial or viral infections.


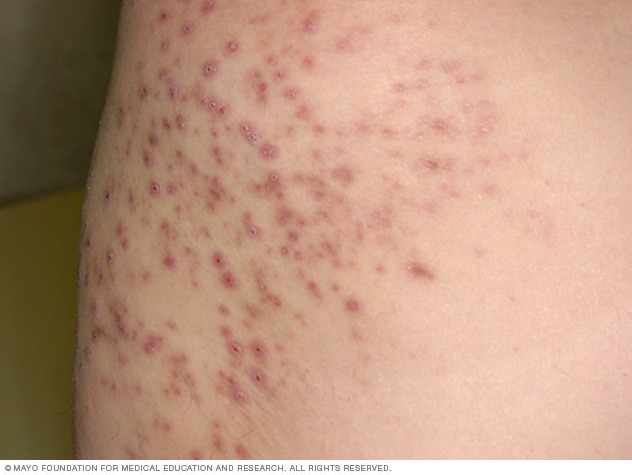


Source: [MayoClinic.org](https://www.mayoclinic.org/diseases-conditions/folliculitis/symptoms-causes/syc-20361634)

Copyright: Mayo Foundation for Medical Education and Research.

**Based on the case description above, what do you feel is the most likely diagnosis?**

**What treatment strategy would you recommend and/or prescribe?**

**10. Case Vignette 10 (OMIT FROM ANALYSIS AFTER CONSULTATION WITH EXPERTS)**

A 26-month old presented with a history of high-grade fever, irritability, reduced oral intake and lesions on the face over the past 4 days. Prior to onset of symptoms, the child reported being generally well. While under outpatient care, the child became increasingly short of breath, swollen right half of face and chest retractions resulting in admission to an intensive care unit. Past medical history revealed antiretroviral treatment for immunocompromised state. Family history revealed mother’s diagnosis of HIV/AIDS. Current vital signs showed a fever of 102 F, >130 beats/minute heart rate and 32 breaths/minute respiratory rate. Physical examination revealed swollen eyes with redness of right eye with conjunctiva. Consulting ophthalmologist did not notice vision abnormalities. Auscultation of lungs revealed clear lung fields, and cardiac examination yielded normal sinus rhythm. The child vaccinations are up to date with the current immunization schedule. The child attends day care and increases exposures to common pathogens.

**
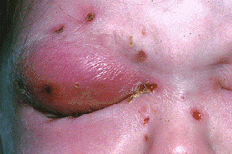
**

Source: Jonathan Trobe, M.D. - University of Michigan Kellogg Eye Center

**Based on the case description above, what do you feel is the most likely diagnosis?**

**What treatment strategy would you recommend and/or prescribe?**

**Module 3: Practice Characteristics**

*To be presented only if and after respondent completes Module 2.*

1. **Which of the following categories BEST describes your employment at your main practice location (i.e., the practice location where you spend the most hours per week)? Select ONE answer.**

| 1 | Full- or part-owner of a Provider practice |
| --- | --- |
| 2 | Employee of a Provider-owned practice |
| 3 | Employee of a large medical group |
| 4 | Employee of a group or staff model HMO |
| 5 | Employee of a university hospital or clinic |
| 6 | Employee of a hospital or clinic not associated with a university (including community health clinics) |
| 7 | Other, please specify:_________________________ |
| 8 | I prefer not to answer |

1. **Is your main practice location in a single specialty or multi-specialty setting? Select ONE answer.**

| 1 | Single specialty |
| --- | --- |
| 2 | Multi-specialty |
| 3 | Other, please specify: _________________________ |
| 4 | I prefer not to answer |

1. **During a typical month, how many pediatric patients do you treat for suspected varicella?**

| 1 | None |
| --- | --- |
| 2 | 1-4 patients |
| 3 | 5-10 patients |
| 4 | 11-20 patients |
| 5 | 21-50 patients |
| 6 | More than 50 patients |
| 7 | Don’t know/Not sure |
| 8 | I prefer not to answer |

1. **During a typical month, how many patients do you prescribe antibiotics for treatment associated with varicella-related infection?**

| 1 | None |
| --- | --- |
| 2 | 1-4 patients |
| 3 | 5-10 patients |
| 4 | 11-20 patients |
| 5 | 21-50 patients |
| 6 | More than 50 patients |
| 7 | Don’t know/Not sure |
| 8 | I prefer not to answer |

1. **During a typical month, how many patients do you prescribe antivirals for treatment associated with varicella-related infection?**

| 1 | None |
| --- | --- |
| 2 | 1-4 patients |
| 3 | 5-10 patients |
| 4 | 11-20 patients |
| 5 | 21-50 patients |
| 6 | More than 50 patients |
| 7 | Don’t know/Not sure |
| 8 | I prefer not to answer |

1. **What share of your patients currently does not have any health insurance coverage? Select ONE answer.**

| 1 | Less than 5% |
| --- | --- |
| 2 | 5% to 9% |
| 3 | 10% to 24% |
| 4 | 25% to 49% |
| 5 | 50% or more |
| 7 | Don’t know/Not sure |
| 8 | I prefer not to answer |

1. **What share of your patients is currently on Medicaid? Select ONE answer.**

| 1 | Less than 5% |
| --- | --- |
| 2 | 5% to 9% |
| 3 | 10% to 24% |
| 4 | 25% to 49% |
| 5 | 50% or more |
| 6 | Don’t know/Not sure |
| 7 | I prefer not to answer |

**Module 4: Demographics**

1. **What is your gender?**

| 1 | Male |
| --- | --- |
| 2 | Female |
| 3 | Other |
| 4 | I prefer not to answer |

1. **What is your age today?**

| 1 | Drop down menu ‘1-115’ |
| --- | --- |
| 2 | I prefer not to answer |

1. **What is your race? Select ONE answer.**

| 1 | American Indian or Alaska Native |
| --- | --- |
| 2 | Asian |
| 3 | Black or African American |
| 4 | Native Hawaiian or Other Pacific Islander |
| 5 | White |
| 6 | Two or more races |
| 7 | Other (please specify) |
| 8 | Don’t know/Not sure |
| 9 | I prefer not to answer |

1. **What is your ethnicity? Select ONE answer.**

| 1 | Hispanic or Latino |
| --- | --- |
| 2 | Not Hispanic or Latino |
| 3 | Don’t know/Not sure |
| 4 | I prefer not to answer |

1. **What are the first 3 digits of the zip code in the location of your primary place of practice?**

| 1 | Numeric 3 digit text box |
| --- | --- |
| 2 | I prefer not to answer |

1. **Do you, as an individual, currently have an affiliation with a medical school, such as an adjunct, clinical, or other faculty appointment?**

| 1 | Yes |
| --- | --- |
| 2 | No |
| 3 | I prefer not to answer |

1. **In what state is your medical degree, nursing, or other advanced degree from?**

| 1 | Drop-down menu of 50 states |
| --- | --- |
| 2 | None of the above |
| 3 | Don’t know/Not sure |
| 4 | I prefer not to answer |

1. **In what year did you graduate from medical school or a nurse practitioner’s program?**

| 1 | Drop down menu ‘1910-2019’ |
| --- | --- |
| 2 | I did not graduate from medical school or nurse practitioner’s program |
| 3 | I prefer not to answer |

Thank you for participating in this survey. We appreciate your time and your responses are valuable.

1. Kimberlin DW. Red Book: 2018-2021 report of the committee on infectious diseases: American academy of pediatrics; 2018. p. 831-842.
