# Supplementary material for: Recognition & management of varicella infections and accuracy of antimicrobial recommendations: Case vignettes study in the US
Source: PLoS One. 2022 Jun 24;17(6):e0269596. doi: 10.1371/journal.pone.0269596 (PMC9231738; doi:10.1371/journal.pone.0269596)
Supplement: Attachment — Submitted filename: Review PONE-D-21-13847.docx [file pone.0269596.s002.docx]

Review PONE-D-21-13847

Intro –page 3, it would be useful to have some case numbers so that the reader could appreciate the absolute reductions along with the percentage drops – at least for conditions with appreciable morbidity (ie hospitalizations, pneumonia, encephalitis)

Is there a more recent case estimate than 2014?

It would be useful to know if any specific qualitative methodology helped to guideline the number/section of cases. Why 9:1 – that seem overly obvious.

Page 6. I would state that the removed varicella case was excluded from table 1.

Did a lot of people struggle with vignette #7? I would think that the correct answer would be hospitalization and antivirals +/- antibiotics for a child with a severe complication of VZV and underlying lung disease.

Table 3. I think should it be NP or APP rather than nurse?

Page 12. What reference are you using to define complicated vs uncomplicated VZV? #4 is really challenging as a in many infectious disease states (ie, complicated UTI or complicated Staph aureus bacteremia) the “complicated” can be defined by EITHER the disease state OR the host being complicated. It is easy in #4 to see how the respondents may have considered a patient on an undefined amount of steroids to be an immunocompromised host and therefore “complicated” even if the presentation itself was not complicated. The fact that >50% called this case complicated suggest that the definitions chosen by the authors may not reflect what is considered complicated in clinical practice.

The following phrase is unclear “Among those who did not recognize varicella infection correctly across all vignettes (n=254),” as there were only 153 respondents. I think this means among those vignettes answered incorrectly? 153*8=1224; 254/1224=0.2 (20% or 254 incidents where varicella was incorrectly identified)

Although the table 4 says only 970 case (79.2%) of cases were correctly identified as varicella.

Lots of numbers are reported but there are really too many to take in.

The most interesting finding is that 20% of varicella cases were missed and 40% of complicated cases were misdiagnosed (if I interpreted this correctly).

This sentence is not clear “Across all uncomplicated vignettes, respondents licensed in the pre-vaccination era were more likely to recognize the case as varicella (94.6%) compared to those licensed in later years (83.3%).” Maybe move the ALL? “Across –all- uncomplicated vignettes, respondents licensed in the pre-vaccination era were more likely to recognize ALL cases as varicella (94.6%) compared to those licensed in later years (83.3%).

So <1996, 5% of uncomplicated cases and 29% of uncomplicated cases were misdiagnosed.

>1996, 23% of uncomplicated cases and 41% of complicated cases were misdiagnosed.

The figures are missing critical information. How the percentages were calculated should be self-evidence when one looks at a figure rather than having to go back to the text to figure out what the authors were using to calculate the percentages. For example, looking at the figure 1b, it is difficult to know whether labs were offered every time and selected incorrectly 100% percent of those times or whether every respondent selected it incorrectly at least once.

Again, I think presenting the #/% missed diagnoses rather than the correct diagnoses would have a great impact on the reader.

Page 13. I would try to avoid “-- were less likely – incorrectly” in the same sentence. That is really hard to read. Consider removing, less likely.

I would probably just write out VPD since it was only used 3 times and it was not a common abbreviation for me.

Perhaps leave more in the tables and put less in text.

What are the really important takeaways?

Was it a few providers who were messing up a lot or many providers missing a few cases?

Was it specific cases or interventions that providers often got wrong?

Which of these wrong selections have critical interventions?

-- Failure to diagnosis uncomplicated cases 🡪 1) failure to quarantine/report/contact track 2) unnecessary antimicrobials/labs

-- Failure to recognize and provide antivirals/antibiotics to complicated cases 🡪 possible life-threating infection

It would be interesting to know whether these same providers would think about quarantine/isolation/reporting/contact tracking (aerosol transmission), but I’m sure that was beyond the scope of this study.
