# Supplementary material for: Recognition & management of varicella infections and accuracy of antimicrobial recommendations: Case vignettes study in the US
Source: PLoS One. 2022 Jun 24;17(6):e0269596. doi: 10.1371/journal.pone.0269596 (PMC9231738; doi:10.1371/journal.pone.0269596)
Supplement: Attachment — Submitted filename: Response to reviewers 5.20.22.docx [file pone.0269596.s004.docx]

**Review Comments to the Author & Responses**

Thank you to the reviewers for taking time to review the paper thoroughly and providing such insightful comments. Please see our responses below. We hope that we have addressed them to your satisfaction.

Best regards,

Authors

------------------------------------------------------------------------------------------------------------------------------------------

**Reviewer #2:**

1. Table 4 Vignette 7 – the word pneumonia is missing

**Response:** The word “pneumonia” has been added to the box in column one.

2. Table 5 – should include the baseline comparison of what is the guideline recommended antiviral/antibiotic use

**Response:** The table has been replaced with a new table that includes baseline comparisons for full context.

3. Second to last sentence of results – remove word recommendations

**Response:** The word “recommendations” has been removed from the relevant sentence.

4. Last sentence of results – rewrite sentence to avoid “less incorrectly” which is confusing

**Response:** The sentence has been reworded and now reads as follows: “Conversely, antivirals were incorrectly recommended less often by HCPs licensed earlier (63.9%) compared to those licensed later (72.1%).”

5. Third sentence of discussion needs to be rewritten for clarity

**Response:** The sentence has been reworded and now reads as follows: “*However, vaccine hesitancy (15) and delays in routine pediatric vaccination due to COVID-19 (11) has impacted the vaccination patterns and coverage rates. It is important to accurately diagnose and manage pediatric infectious diseases such as varicella.”*
